# Pathological mechanisms of long non-coding RNA FOXD2-AS1 promoting osteosarcoma mediated by the COI-1/OCN/Runx-2 signaling pathway

**DOI:** 10.3389/fmed.2026.1664094

**Published:** 2026-09-30

**Authors:** Zhipeng Ren, Liandong Deng, Jie Sun, Yuxiang Kang, Hejun Zhao

**Affiliations:** 1 Department of Orthopaedics, Tianjin Hospital, Tianjin, China; 2 Department of Orthopaedics, Tianjin Hospital, Tianjin University, Tianjin, China; 3 Department of Polymer Science and Technology, School of Chemical Engineering and Technology, Tianjin University, Tianjin, China; 4 Department of Endocrinology and Metabolism, Tianjin First Central Hospital, Tianjin, China; 5 Department of Endocrinology and Metabolism, Tianjin First Central Hospital, Affiliated Hospital of Nankai University, Tianjin, China

**Keywords:** FOXD2-AS1, hydrogel, long non-coding RNA, osteosarcoma, pathological mechanisms

## Abstract

**Introduction:**

Osteosarcoma (OS) is the most common primary malignant bone tumor in children and adolescents. Despite three decades of research efforts, no significant improvement in patient survival rates has been achieved, highlighting the urgent need for in-depth investigation into its pathogenesis to develop novel therapeutic strategies and more effective drugs. FOXD2-AS1, a long non-coding RNA, has been implicated in the prognosis and survival of various cancers, but its role in OS remains underexplored. This study therefore aimed to investigate the functional involvement of FOXD2-AS1 in OS progression and to evaluate a biocompatible delivery system for its in vitro and in vivo application.

**Methods:**

We synthesized mPEG-PDPA hydrogel via free radical polymerization of 2-(diisopropylamino)ethyl methacrylate (PDA) and polyethylene glycol monomethyl ether acrylate (mPEG-AC), and employed this hydrogel as a delivery vehicle for FOXD2-AS1. The biocompatibility and safety of the empty hydrogel were first validated through both *in vitro* cell assays and *in vivo* animal experiments. To explore the mechanistic actions of FOXD2-AS1, we performed real-time quantitative PCR and Western blot analyses, and conducted transcriptome profiling to identify downstream pathways and associated genes.

**Results and Discussion:**

Our results demonstrated that the mPEG-PDPA hydrogel itself was non-toxic and reliable as a delivery vehicle. Functionally, FOXD2-AS1 promoted alkaline phosphatase (ALP) release and exacerbated the inflammatory response, thereby accelerating the development and deterioration of OS. Mechanistically, FOXD2-AS1 activated the F-box protein Coronatine Insensitive 1 homolog (COI-1)/OCN/Runx-2 signaling pathway, as confirmed by qPCR and Western blot. Transcriptome analysis further revealed that this process involved changes in cell migration, apoptosis, and cell cycle regulation, with CPS1-IT1, ANKRD10-IT1, TBC1D3P1-DHX40P1, and RGS5 identified as key associated genes. Collectively, our findings provide new insights into the pathological mechanisms of OS and offer valuable data support for future therapeutic strategy development.

## 1 Introduction

Osteosarcoma (OS) as the most common primary malignant bone tumor in children and adolescents which originates from malignant mesenchymal cells (1). The overall 5-year survival rate for OS is only 55% due to the early onset of metastases and the high rate of recurrence and chemotherapy resistance, and less than 20% for patients with combined lung metastases (2). Currently, the main treatment options for osteosarcoma are preoperative (neoadjuvant chemotherapy) and postoperative polychemotherapy (adjuvant chemotherapy) and surgical reconstruction techniques, with chemotherapy regimens still relying on drugs similar to those used in the 1980s and facing the limitations of chemotherapy resistance (3, 4). However, the survival rate of OS has not improved over the last three decades of endeavor (5). Hence, there is still a need to develop new therapeutic strategies and highly effective drugs to obtain better outcomes in OS.

Cancer being a complex disease, essentially characterized by mutations in genes controlling multiple features of the disease, including evasion of programmed cell death, induction of genomic instability and mutations, and proliferative signaling (6, 7). In the past two decades numerous clinical trials based on gene therapy have been conducted (8). Recent studies have suggested that RNA interference (RNAi) demonstrates favorable applications in the treatment of various diseases encompassing cancer (9), cardiovascular diseases (10) and metabolic disorders (11). Long non-coding RNAs (LncRNAs) are endogenous RNA molecules with transcript lengths longer than 200 nucleotides, which encode no proteins or only very short polypeptides (12). LncRNAs regulate important processes including tumorigenesis, growth, apoptosis and metastasis through epigenetic modifications, transcriptional activation, and regulation of protein function (13, 14). FOXD2-AS1 is a LncRNA of 2527bp in a cluster of genes located in the telomere region of the human chromosome at the 1p33 position. FOXD2-AS1 is aberrantly expressed in various malignant tumors including gastric, lung, colorectal and hepatocellular carcinomas, and its dysregulated expression is closely associated with patient survival and prognosis (15, 16). Our previous study showed that knockdown of LncRNA FOXD2-AS1 resulted in decreased proliferation and migration and increased apoptosis of OS; moreover, analysis of clinical information of OS patients revealed that FOXD2-AS1 was an independent risk factor for poor prognosis of OS (17). These findings reveal that FOXD2-AS1 plays an important role in the development and progression of OS. Therefore, it is necessary to elucidate the biological mechanisms underlying the promotion of OS progression via FOXD2-AS1.

Local delivery techniques allow better tolerance of high doses of locally administered drugs than systemic delivery, which is usually complicated by side effects such as nephrotoxicity, hepatotoxicity, and hepatotoxicity (18). Combining therapeutic compounds with bone affinity with nanocarriers can effectively improve drug delivery (19). The 2-(diisopropylamino)ethyl methacrylate (PDA) and polyethylene glycol monomethyl ether acrylate (mPEG-AC) are used as pH-responsive moieties for gene delivery vectors to achieve selective controlled release of genes (20, 21). The amphiphilic block copolymer methoxy-poly(ethylene glycol)-block-poly(2-(diisopropylamino)ethyl methacrylate) (mPEG-b-PDPA) was selected as the foundational material for our delivery system. Its utility stems from the sharp pH-responsive behavior of the PDPA block. The tertiary amines in PDPA are deprotonated and hydrophobic at physiological pH (≈7.4), driving the self-assembly of micelles or hydrogels. In the slightly acidic tumor microenvironment (pH ≈ 6.5–6.8), these amines become protonated, rendering the PDPA block hydrophilic and causing the nanostructure to disassemble. This mechanism allows for stable payload encapsulation during systemic circulation and triggered, site-specific release within tumors. The hydrophilic mPEG corona provides steric stabilization, reduces protein adsorption, and extends blood circulation time.

Thus, in this study, pH-responsive DPA was polymerized with mPEG-AC in free radical solution to form an amphiphilic grafted copolymer (mPEG-PDPA hydrogel), which was used as a delivery vector for LncRNA FOXD2-AS1. Herein, we probed the complex site of mPEG-PDPA and LncRNA FOXD2-AS1 by Fourier transform infrared spectroscopy and analyzed the stability of the complex by zeta potential and size. The biotoxicity of mPEG-PDPA against MG63 osteosarcoma cells was tested by *in vitro* cellular assays. Next, the molecular mechanism of FOXD2-AS1 deterioration in osteosarcoma was analyzed by *in vivo* and *in vitro* experiments of mPEG-PDPA-RNA administration combined with immunohistochemistry, hematoxylin-eosin staining, western blot, RT-qPCR and transcriptomics. This study provided data support for subsequent investigation of the mechanism of osteosarcoma development mediated by FOXD2-AS1.

## 2 Materials and methods

### 2.1 Materials

All chemicals were purchased from Sigma-Aldrich and were not further purified before use. Amphiphilic grafted copolymer mPEG-PDPA hydrogel was prepared by free radical polymerization reaction in N,N-Dimethylformamide (DMF) solution, using 2,2-Azobisisobutyronitrile (AIBN) as initiator, polyethylene glycol monomethyl ether acrylate (mPEG-AC) as hydrophilic monomer and 2-(diisopropylamino)ethyl methacrylate (PDA) as hydrophobic monomer. Details of the synthesis of mPEG-PDPA were given in Supplementary Text S1.

### 2.2 Synthesis of mPEG-PDPA-loaded FOXD2-AS1 (mPEG-PDPA-RNA)

LncRNA FOXD2-AS1 was loaded onto mPEG-PDPA hydrogels by gel electrophoresis (22). Briefly, a 1% agarose gel (Agarose Gel) containing the nucleic acid dye was prepared and set aside. Subsequently, mPEG-PDPA was mixed with LncRNA FOXD2-AS1 at different 100:5 mass ratios and incubated for 20 min with 400 ng of LncRNA FOXD2-AS1 in each well. Finally, 6× loading buffer was added and mixed well, and the loading was completed by electrophoresis at a voltage of 120 V for 20 min.

### 2.3 Optimized synthesis and characterization of mPEG-b-PDPA copolymers

mPEG-b-PDPA copolymers were synthesized via reversible addition-fragmentation chain-transfer (RAFT) polymerization using mPEG as a macro-chain transfer agent, as previously described with modifications (23). Briefly, mPEG, 2-(diisopropylamino)ethyl methacrylate (DPA), and azobisisobutyronitrile (AIBN) initiator were dissolved in anhydrous DMF in a Schlenk tube under a nitrogen atmosphere. The molar feed ratio of mPEG to DPA was varied (e.g., 1:50, 1:60, 1:70) to obtain polymers with different PDPA block lengths. The reaction proceeded at 73 °C for 7 h. The purified copolymers were characterized by dynamic light scattering (DLS) to evaluate their hydrodynamic diameter and colloidal stability.

### 2.4 Characterization

Scanning electron microscopy (SEM, TESCAN MIRA-4, Czech Republic) was used to observe the morphology of the mPEG-PDPA-RNA materials. Nanoparticle size and zeta potential analyzer (DLS, Brookhaven, USA) was used to test the zeta potential and size of the materials. Fourier transform infrared spectroscopy (FTIR, ThermoFisher, USA) was used to analyze the functional groups on the surface of the materials.

### 2.5 Cell lines

Osteosarcoma cell lines (MG63, Saos-2) from Type Culture Collection Centre (Shanghai, China) were selected for *in vivo* experiments. Cells were dispersed in cell complete culture medium: 90% DMEM, 10% FBS, and 1% penicillin-streptomycin solution, and incubated at 37 °C in an incubator (5% carbon dioxide and 70%–80% humidity). Ethical approval for this study was obtained from the Institutional Review Board of Medical Ethics Committee at Tianjin Hospital (2021-178), and all biological experiments were conducted in strict compliance with the guidelines for the use of laboratory animals established by the National Institutes of Health (NIH).

### 2.6 Cell viability assay (CCK-8)

Cell viability was assessed using the Cell Counting Kit-8 (CCK-8). MG-63 cells were seeded in 96-well plates and incubated overnight. After treatment, 10 µL of CCK-8 reagent was added to each well. Following a 2-h incubation at 37 °C, the absorbance at 450 nm was measured using a microplate reader. The absorbance values from wells containing only medium were used as blank controls. Cell viability for each treatment group was expressed as a percentage relative to the untreated control cells.

### 2.7 Cell cycle assay

Cell cycle assays were performed using the Cell Cycle and Apoptosis Assay Kit (C1052, Beyotime) and were performed in strict accordance with the supplier's guidelines. Briefly, cells were treated with mPEG-PDPA at concentrations of 0, 65, 125, 250, 500 and 1,000 μg/mL for 24 h. After treatment, cell cycle analysis was performed according to the supplier's requirements.

### 2.8 Cell apoptosis assay

A flow cytometry was used to analyze the effect of mPEG-PDPA on apoptosis of MG63 osteosarcoma cells. MG63 cell samples were treated with mPEG-PDPA at concentrations of 0, 65, 125, 250, 500 and 1,000 μg/mL for 24 h. Cell samples were stained with Annexin V-FITC Apoptosis Detection Kit (C1062L, Beyotime) using Annexin-conjugated Fluorescein Isothiocyanate (FITC) and propidium iodide (PI). The samples were then immediately analyzed using a flow cytometer (BD FACS Calibur) and the results were measured using BD Cell Quest Pro Ver.6.0 software.

### 2.9 Cell scratch assay

Cell migration was assessed using a cell scratch assay. MG-63 cells were seeded in 6-well plates and grown to confluence. A uniform scratch was created across the cell monolayer using a sterile 200 μL pipette tip. After washing with PBS to remove detached cells, fresh medium containing the respective treatments was added. Images of the scratch were captured at the time of wounding (0 h) and after a defined incubation period (e.g., 24 h) using an inverted phase-contrast microscope. The migration distance was quantified by measuring the change in wound width at multiple predetermined points using ImageJ software (National Institutes of Health, USA).

### 2.10. MTT assay

MTT analyses were performed using the MTT Cell Proliferation and Cytotoxicity Assay Kit (C0009S, Beyotime). The operation was performed strictly according to the supplier's instructions.

### 2.11. Animal and osteosarcoma modelling

According to our previously reported method (24), 5 × 10^6^ MG63 cells were suspended in 100 μL PBS and injected into the abdomen of 4–5 weeks, male BALB/c nude mice (Beijing Charles River Animal Breeding Company Limited, animal certificate: SYXK (Shandong) 2017-0022). After 6 weeks of observation, the width and length of osteosarcomas were recorded and tumors were weighed after the mice were executed. Tumor growth was monitored by measuring the length (*L*) and width (*W*) of each tumor using a digital caliper at designated time points: 0, 3, 7, 14, 21, 28, 35, and 42 days post-treatment initiation. The tumor volume (TV) was calculated according to the formula: TV = (*L* × *W*^2^)/2 (5). On day 42, mice were anesthetized with isoflurane (1.5% isoflurane in 98.5% O_2_) and subjected to *in vivo* bioluminescence imaging using an IVIS Spectrum imaging system (PerkinElmer, USA) following intraperitoneal injection of D-luciferin (150 mg/kg) to assess the tumor burden and metastatic dissemination (25). After imaging, the mice were euthanized by cervical dislocation under deep isoflurane anesthesia, and the tumors were immediately excised, photographed, weighed, and either fixed in 4% paraformaldehyde for histological analysis or snap-frozen in liquid nitrogen for molecular analyses. All animal procedures were performed in accordance with the ARRIVE guidelines and approved by the Institutional Review Board of Medical Ethics Committee at Tianjin Hospital (2021-178).

### 2.12. Hematoxylin-eosin (HE) staining

Paraffin sections (3 μm) were deparaffinized and rehydrated and stained using hematoxylin and eosin stains according to the method reported by previous researchers (26).

### 2.13. Immunohistochemistry assay

Paraffin sections were dewaxed and rehydrated, and antigen repair was performed in a microwave oven filled with citric acid antigen repair buffer (pH: 6.0) for 5 min each on medium-high and medium-high heat. After returning to room temperature, endogenous peroxidase was inactivated by dropwise addition of endogenous peroxidase blocker solution for 10 min and incubated with primary antibody at 4 °C overnight. Sections were incubated with primary antibody overnight at 4 °C. Drops were incubated with horseradish peroxidase-coupled goat anti-rabbit IgG secondary antibody (1:100 dilution) for 60 min at 37 °C. Color was developed using DAB chromogenic solution. Hematoxylin reduction was followed by alcohol gradient dehydration. Sections were cleared in xylene and sealed with neutral gum and observed under a microscope. Information on the antibodies used in this test was given in Supplementary Table S1.

### 2.14. Alkaline phosphatase (ALP) analysis

Alkaline phosphatase (ALP) analysis was performed using the ALP assay kit (A059-2) from Nanjing Jiancheng Bioengineering Institute. Alkaline phosphatase breaks down disodium benzene phosphate and produces free phenol and phosphoric acid. The analysis was performed in strict accordance with the supplier's instructions. The principle of this kit is: phenol interacts with 4-aminoantipyrine in alkaline solution and is oxidized by potassium ferricyanide to produce red quinone derivatives, and the absorbance of the samples after the display was detected at 520 nm for calculation of ALP activity.

### 2.15. RNA extraction and RT-qPCR

Total RNA was extracted from tissue cells with Trizol reagent and reverse transcribed into cDNA. RNA content and purity were measured by UV spectrophotometer (optical density ratio of RNA at 260 nm and 280 nm between 1.8 and 2.0). The integrity was identified by 1% agarose gel electrophoresis (ratio of 28S and 18S RNA bands ≥2.0). Literature was searched and Omiga 2.0 software was applied to design primer sequences for ALP, F-box protein coronatine-insensitive 1 (COI-1), osteocalcin (OCN) and runt-related transcription factor 2 (Runx-2) genes. Gene amplification was performed with a PCR instrument (Applied Biosystems). The above RNAs were detected using the RT-qPCR kit from Tengen. Statistical analysis was done with the mean value of the results of 3 experiments. The primer sequences used in this study are given in Supplementary Table S2.

### 2.16. Western blot assay

Western blotting was used to detect changes in the expression of Collagen I, Osteocalcin and Runx2 proteins. Mouse tissues were lysed with RIPA cell lysate on ice in the presence of a tissue homogenizer, and proteins were extracted by centrifugation at low temperature for 5 min, and protein was quantified by BCA method (Beyotime, P0011). Add protein uploading buffer and denature at 100 °C for 5 min. 60 μg of protein per well was uploaded and electrophoresed on 10% SDS-polyacrylamide gel. The electrophoresis products were transferred to PVDF membrane. The membrane was closed with 5% skimmed milk powder for 2 h. The primary antibody was added overnight at 4 °C. The membrane was washed with TBST, and the secondary antibody was conjugated at 37 °C for 2 h. ECL Plus Ultrasensitive Luminescent Solution (Beyotime, P0018M) was used to develop the image. Images were analyzed by Quantity One Gel Imaging Analysis System and repeated 3 times. Information on the antibodies used in this study is given in Supplementary Table S3.

### 2.17. Transcriptome analyses

Total RNA was extracted and purified from osteosarcoma tissues of each group of osteosarcoma model mice (*n* = 6). NanoDrop spectrophotometer (ThermoFisher, USA) and TapeStation automated electrophoresis system (Agilent, USA) were used to detect the quantity and quality of RNA, respectively. Concomitantly, mRNA was isolated from total RNA by polyt oligo magnetic beads. sequencing libraries were generated using the VAHTS mRNA-seq v2 library preparation kit from Illumina (Vazyme Biotech, China). These libraries were sequenced on the Illumina NovaSeq platform to generate 150 bp paired-end reads. All manipulations and analytical procedures were performed in strict accordance with the manufacturer's instructions.

Raw data in fastq format were first processed for primary quality control. Fragments mapped per kilobase exon per million fragments (FPKM) in each sample were calculated based on the length of the fragment and the count of reads mapped to that fragment. Differential expression analyses were performed using the DEGSeq R software package (1.20.0) for the control and mPEG-PDPA-RNA material treatment groups, using a *P* value of <0.05 as the cut-off value for significantly differentially expressed genes (DEGs), and gene ontology (GO) and kyoto encyclopedia of genes and genomes (KEGG) pathway enrichment analyses for DEGs (27). In addition, empowering the development of genomics expertise (EDGE) analysis between groups was performed using the edgeR software package (4.2.0).

### 2.18. Statistical analysis

Without special emphasis, all cell experiments were repeated more than three times and all animal experiments were repeated more than six times. Experimental results were expressed as mean ± variance, and all data were analyzed using SPSS 27.0 statistical software, using one-way ANOVA and LSD-t test for differences between groups. Herein, *p-value <0.05 was considered statistically different between groups.

## 3 Results

### 3.1 Binding sites of mPEG-PDPA and LncRNA and stability of the complexes

The morphology of the prepared mPEG-PDPA and mPEG-PDPA-RNA was observed by scanning electron microscopy (SEM). The surface of pristine mPEG-PDPA was rough, accompanied by an undulating structure (Figure 1a), which increased the combinatorial sites of the material for LncRNA. After complexation with LncRNA, the surface of the prepared mPEG-PDPA-RNA was smooth and the rough structure was hardly visible (Figure 1b). These indicated that the complexation of mPEG-PDPA with RNA resulted in the disappearance of the rough surface. Furtherment, Fourier transform infrared spectroscopy (FTIR) was used to detect changes in the functional groups of mPEG-PDPA hydrogels before and after LncRNA loading (Figure 1c). The results showed that after complexation, the peaks of mPEG-PDPA-RNA located at 3,427 and 2,716 cm^−1^ decayed, indicating that -OH is the main active site when mPEG-PDPA and LncRNA bind. In addition, the IR peaks located at 1,467 and 1,732 cm^−1^ in mPEG-PDPA-RNA could be assigned to RNA nucleobases, whereas the IR peak at 962 cm^−1^ was attributed to the phosphate backbone vibration (νP-O) (28). These results indicated that FOXD2-AS1 LncRNA was successfully loaded on mPEG-PDPA hydrogels and that -OH was the main active site for the binding of mPEG-PDPA and LncRNA (29).

**Figure 1:**
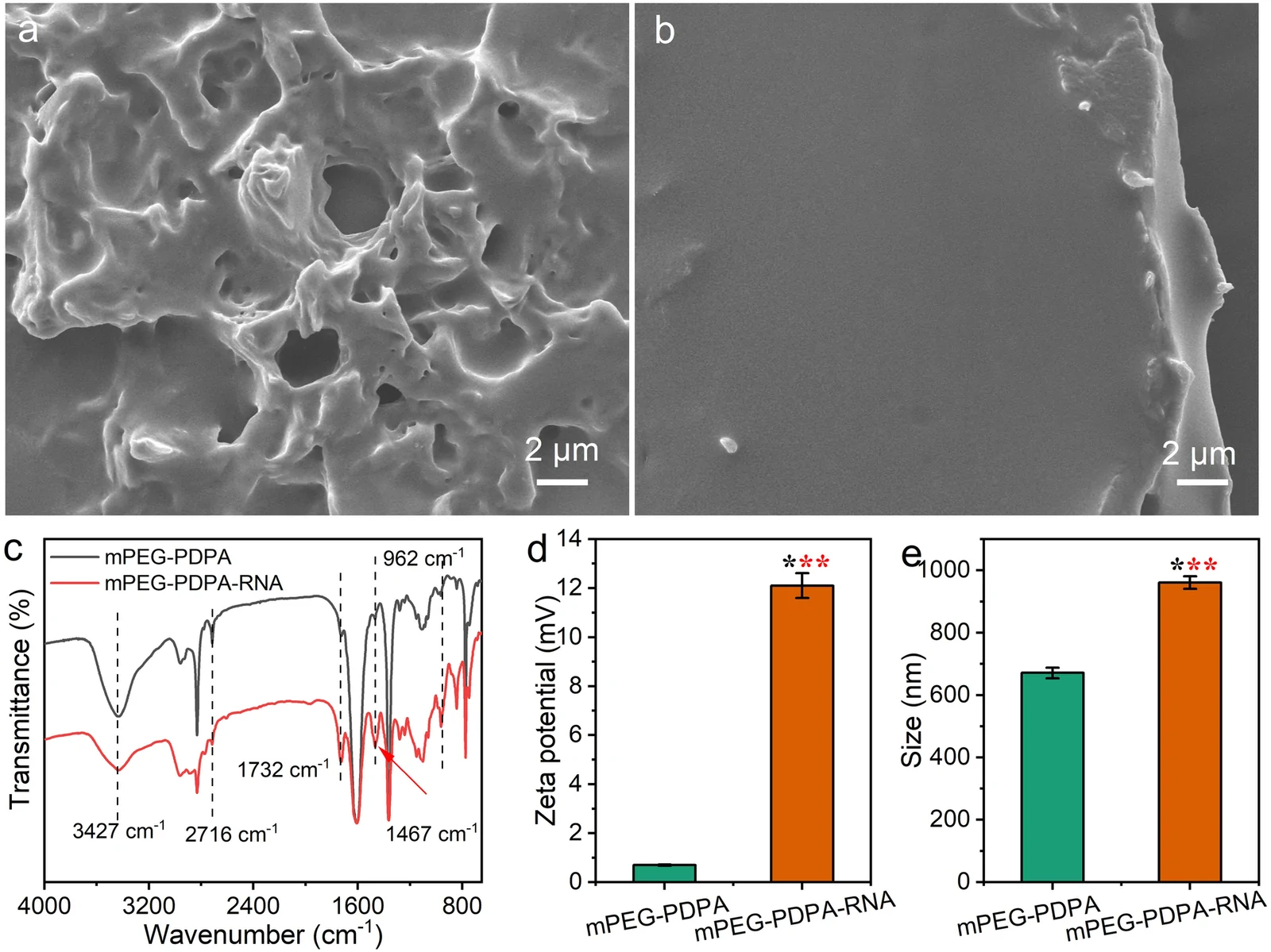
SEM images of **(a)** mPEG-PDPA and **(b)** mPEG-PDPA-RNA. **(c)** FTIR spectra of mPEG-PDPA-RNA. **(d)** Zeta potential and **(e)** size of mPEG-PDPA-RNA measured by dynamic light scattering (DLS). Here, *** represents *p*-value less than 0.001. The red and black stars in the figures represent the same statistical significance levels, with the color difference serving only as a visual aid to highlight the increasing levels of significance.

Dynamic light scattering (DLS) was employed to analyze the changes in zeta potential and size of mPEG-PDPA hydrogels before and after LncRNA loading. As shown in Figure 1d, the zeta potential values of the complexed mPEG-PDPA-RNA hydrogels were significantly enhanced after LncRNA loading (*p* < 0.001), indicating that mPEG-PDPA-RNA has strong stability. In addition, the particle size of mPEG-PDPA-RNA increased significantly (*p* < 0.001) due to the complexation with LncRNA (Figure 1e). To identify the most stable formulation for subsequent experiments, the colloidal stability of mPEG-PDPA copolymers with different PDPA block lengths (molar feed ratios of 1:50, 1:60, and 1:70) was further evaluated by DLS after RNA loading. As shown in Supplementary Figure S1, the mPEG-PDPA-1/60 formulation exhibited no significant changes in hydrodynamic diameter or zeta potential over an 8 h period, indicating superior colloidal stability compared to the other ratios. Furthermore, the RNA release profile of the mPEG-PDPA-1/60 formulation was assessed under physiological conditions (pH: 7.4, 37 °C). The results demonstrated that approximately 35% of the loaded RNA was released within 8 h (Supplementary Figure S2), confirming a sustained release behavior suitable for subsequent biological applications. Therefore, mPEG-PDPA-1/60 was selected for all subsequent *in vitro* and *in vivo* experiments. Unless otherwise specified, “mPEG-PDPA” in the following sections refers to the mPEG-PDPA-1/60 formulation. These results confirmed that mPEG-PDPA after loading LncRNA has strong stability, is not easy to agglomerate, and has reliable potential for application (30).

### 3.2 Almost no cytotoxicity of mPEG-PDPA hydrogels

The effect of the administered dose of mPEG-PDPA on the survival of MG63 osteosarcoma cells was analyzed according to the MTT test (Figure 2a), and the treatment of a gradient of mPEG-PDPA concentration from 0 to 1,000 μg/L for 24 h had almost no effect on the survival of MG63 osteosarcoma cells (*p* > 0.05). Flow cytometric apoptosis assay showed that none of the treatments with each mPEG-PDPA concentration resulted in apoptosis of MG63 osteosarcoma cells (Figure 2b and Supplementary Figure S3). These results indicated that mPEG-PDPA is virtually non-toxic to MG63 osteosarcoma cells and is a highly biophilic LncRNA carrier. As shown by the results of flow cytometric cycle assay, mPEG-PDPA treatment resulted in a significant decrease in the number of G1-phase cells compared to the blank group (*p* < 0.05), and with the increase of the dose the number of S-phase cells gradually started to increase whereas the number of G2-phase cells relatively started to decrease (Figure 2c and Supplementary Figure S4). This suggested that mPEG-PDPA promotes the progression of mitosis in MG63 osteosarcoma cells, further demonstrating that mPEG-PDPA is virtually non-toxic to MG63 cells.

**Figure 2:**
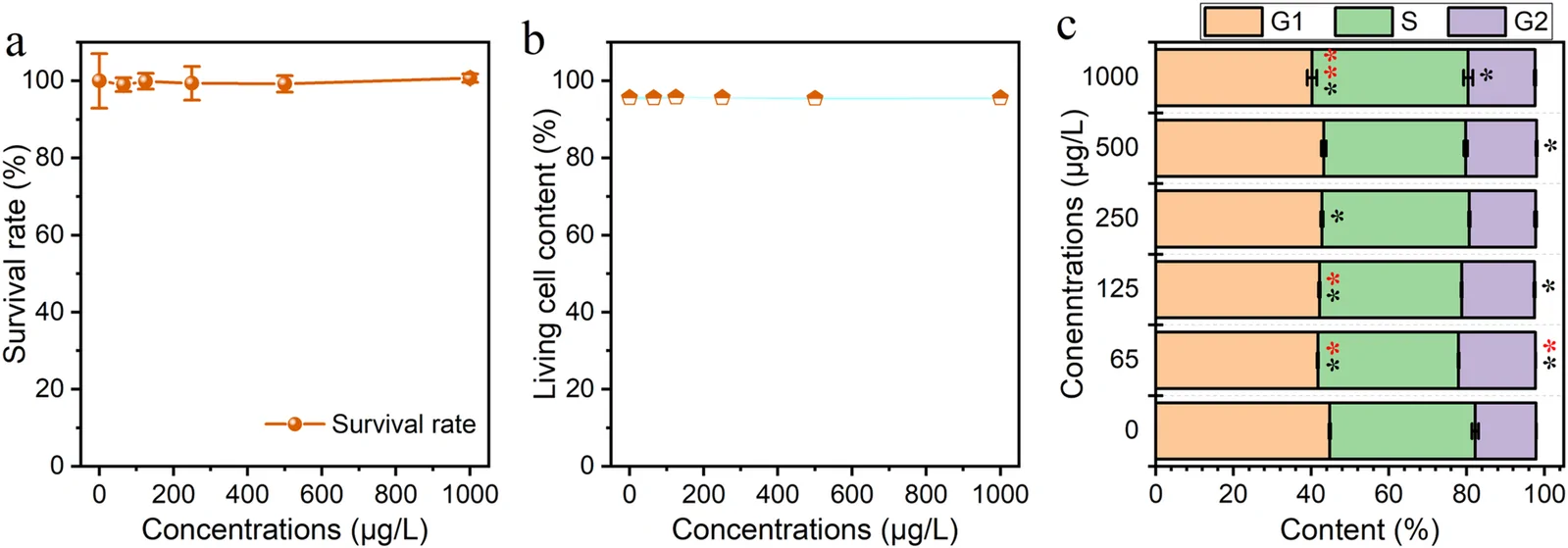
Toxic effects of different doses of mPEG-PDPA on MG63 cells (*n* = 3). **(a)** 24 h survival of MG63 osteosarcoma cells at different mPEG-PDPA doses (MTT assay). **(b)** Proportion of live cells in flow cytometric apoptosis assay. **(c)** Effects of different mPEG-PDPA doses on the cell cycle of MG63 osteosarcoma cells. *, ** and *** represent the results of statistical difference analysis with *p*-values less than 0.05, 0.01 and 0.001, respectively. The red and black stars in the figures represent the same statistical significance levels, with the color difference serving only as a visual aid to highlight the increasing levels of significance.

### 3.3 Accelerated progression of osteosarcoma by mPEG-PDPA-RNA treatment

Serum alkaline phosphatase (ALP) is significantly elevated in osteosarcoma patients compared to healthy individuals, and has been associated with the development of osteosarcoma (31, 32). Therefore, ALP can be considered as a biomarker for this type of tumor progression in the osteosarcoma mouse model. As shown in Figure 3a, our results indicated that mPEG-PDPA-RNA significantly led to the up-regulation of ALP in osteosarcoma levels in osteosarcoma model mice in an *in vivo* assay (*p* < 0.001), whereas mPEG-PDPA had almost no effect on ALP levels (*p* > 0.05). To validate the transcriptomic changes associated with osteogenic differentiation observed in our preliminary RNA-seq screening, we next examined the expression of key marker genes at the mRNA level. First, we confirmed that the elevated serum ALP activity was paralleled by a significant increase in ALP gene expression within tumor tissues, as determined by RT-qPCR. As shown in Figure 3b, the results of real-time quantitative PCR (RT-qPCR) confirmed that the expression of ALP gene was up-regulated in the model mice under the treatment of mPEG-PDPA-RNA (*p* < 0.001), while mPEG-PDPA had almost no effect on the expression of ALP gene (*p* > 0.05).

**Figure 3:**
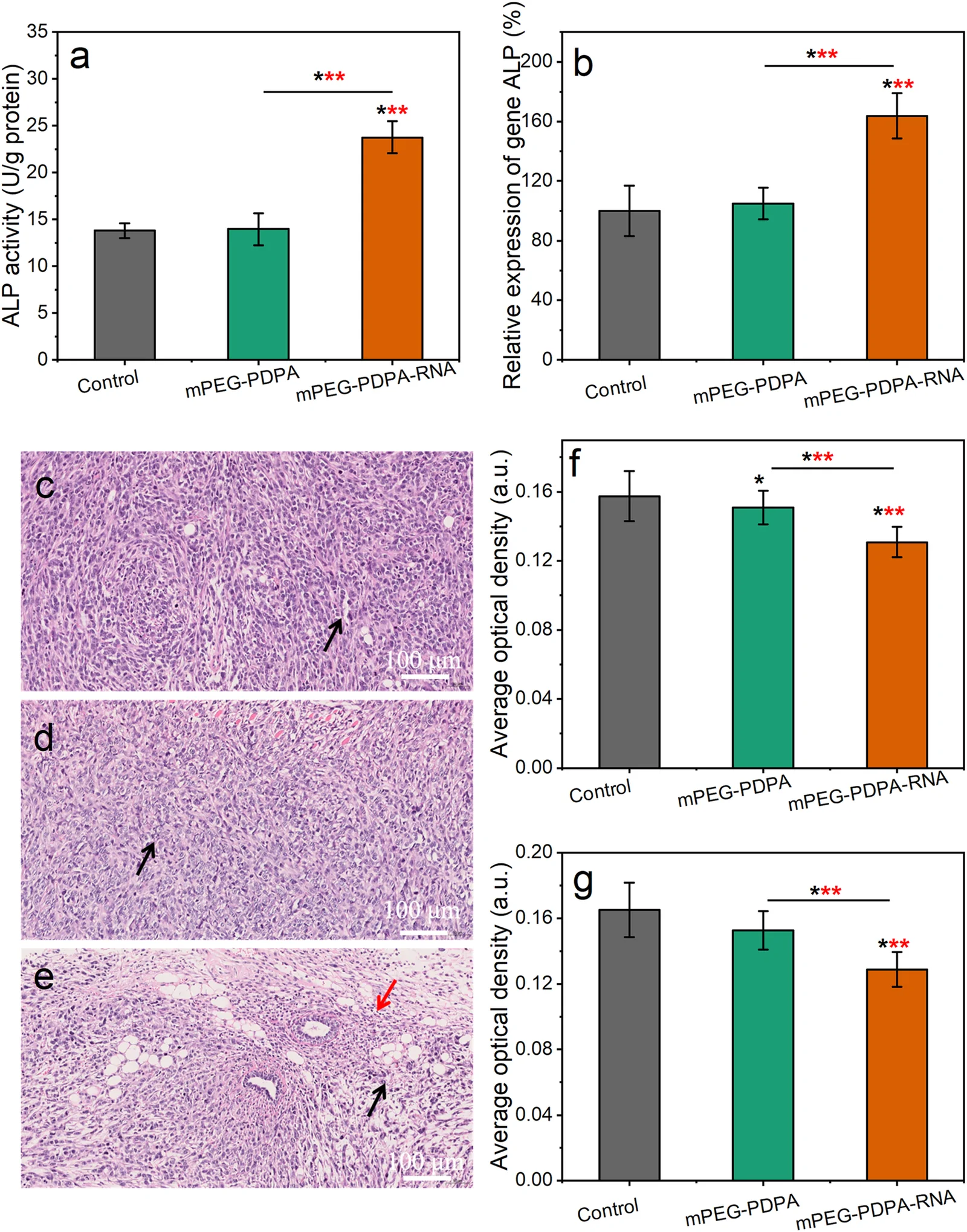
FOXD2-AS1 promotes the expression of alkaline phosphatase (ALP), a typical tumor biomarker, in osteosarcoma in model nude mice (*n* = 6). **(a)** ALP levels and **(b)** relative expression of ALP genes in osteosarcoma. Hematoxylin eosin (HE) staining results of osteosarcoma from model mice in **(c)** control, **(d)** mPEG-PDPA and **(e)** mPEG-PDPA-RNA groups. **(f)** Average optical density (AOD) statistics of CD31 and **(g)** Ki67 immunohistochemical results. Herein, *** represents *p*-value less than 0.001. The red and black stars in the figures represent the same statistical significance levels, with the color difference serving only as a visual aid to highlight the increasing levels of significance.

Hematoxylin eosin (HE) staining results on osteosarcoma showed that the overall structure of the tumor tissue in the control and mPEG-PDPA groups was mildly abnormal, with spindle-shaped tumor cells, oval nuclei, and pathologic karyorrhexis images were seen (shown by the black arrows), and there was no obvious infiltration of inflammatory cells in the tissues (Figures 3c,d). In contrast, as shown in Figure 3e, the overall structure of osteosarcoma tissues in the mPEG-PDPA-RNA group was mildly abnormal and a small number of tumor cells were degenerated and necrotic (shown by black arrows), and a small amount of inflammatory cell infiltration was seen in the tissues (shown by red arrows). These indicated that FOXD2-AS1 exacerbated the inflammatory response of osteosarcoma tissues and induced tumor deterioration. Immunohistochemical results showed that compared with the control group, the mean optical density values of CD31 and Ki67 in the mPEG-PDPA-RNA group underwent a significant reduction (*p* < 0.001), whereas only the reduction of CD31 in the mPEG-PDPA group was statistically different (*p* < 0.05), as shown in Figures 3f,g and Supplementary Figure S6.

The antitumor efficacy of mPEG-PDPA-RNA was further evaluated in the BALB/c nude mouse xenograft model. Tumor volumes were measured at designated time points (0, 3, 7, 14, 21, 28, 35, and 42 days post-treatment initiation) using a digital caliper. As shown in Supplementary Figures S7–S9, mice treated with mPEG-PDPA-RNA exhibited the smallest tumor volume increase throughout the observation period, whereas the control group (PBS) and empty mPEG-PDPA hydrogel group showed rapid and comparable tumor growth. These results demonstrated that delivery of FOXD2-AS1 siRNA via mPEG-PDPA hydrogels significantly suppressed osteosarcoma tumor growth *in vivo*.

### 3.4 Potential molecular mechanism of FOXD2-AS1 promoting osteosarcoma

To evaluate the *in vitro* therapeutic potential of the mPEG-PDPA-RNA complex, we performed a CCK-8 assay. As shown in Supplementary Figure S10, the empty mPEG-PDPA carrier exhibited no significant cytotoxicity compared to the Control group, confirming its biocompatibility. In contrast, the mPEG-PDPA-RNA complex, which delivers siRNA targeting FOXD2-AS1, significantly and dose-dependently reduced cell viability. At the highest concentration tested (2X), cell viability was inhibited to approximately 61% (*p* < 0.001). These results demonstrate that effective knockdown of FOXD2-AS1 using our delivery system directly impairs the proliferation and survival of osteosarcoma cells *in vitro*. As shown in Supplementary Figures S11, S12, silencing FOXD2-AS1 with the mPEG-PDPA-RNA(2X) complex significantly inhibited cell migration. The migration rate was reduced to 25.71 ± 1.50%, compared to 41.92 ± 3.02% in the Control group (*p* < 0.001). The mPEG-PDPA carrier alone had no significant effect (43.99 ± 4.13%). This finding indicates that FOXD2-AS1 is crucial for regulating the migratory capacity of osteosarcoma cells, a key step in metastasis.

To elucidate the molecular mechanisms underlying the observed functional suppression, we analyzed the expression of proteins critical for cancer stemness, EMT, and metastasis. Western blot analysis revealed that silencing FOXD2-AS1 with mPEG-PDPA-RNA(2X) induced a profound shift in the molecular profile of osteosarcoma cells (Supplementary Figures S13, S14). Notably, we observed a significant upregulation of the epithelial adhesion molecule E-Cadherin and a concurrent downregulation of mesenchymal markers (N-Cadherin, Vimentin), the EMT activator Snail, and invasion-promoting enzymes (MMP2, MMP9). Furthermore, the expression of core stemness factors (Nanog, OCT4, SOX2) and the stem cell marker CD133 was markedly decreased, as was the angiogenic factor VEGF. The mPEG-PDPA carrier alone did not alter the expression of any these markers. These data indicate that FOXD2-AS1 is a master regulator of an integrated network driving stemness, EMT, invasion, and angiogenesis in osteosarcoma.

To further validate and pinpoint the key differentially expressed pathway from our RNA-seq analysis, we focused on the osteogenic differentiation axis. RT-qPCR analysis confirmed that the expression of genes encoding F-box protein coronatine-insensitive 1 (COI-1), osteocalcin (OCN), and runt-related transcription factor 2 (Runx-2) was significantly up-regulated in osteosarcoma tissues from mice treated with mPEG-PDPA-RNA (Figures 4a–c). This direct transcriptional validation underscores the activation of the COI-1/OCN/Runx-2 signaling pathway upon FOXD2-AS1 knockdown. Moreover, the expression of F-box protein coronatine-insensitive 1 (COI-1), osteocalcin (OCN) and runt-related transcription factor 2 (Runx-2) genes was significantly up-regulated in mouse osteosarcoma after mPEG-PDPA-RNA treatment (*p* < 0.001), in contrast to mPEG-PDPA which barely affects the expression of these genes (*p* > 0.05). Additionally, the expression of Collagen I, Osteocalcin and Runx-2 proteins in osteosarcoma was analyzed (Figures 4d–g). The results showed that mPEG-PDPA-RNA similarly induced up-regulation of the expression of the relevant proteins in osteosarcoma in model mice (*p* < 0.01), while mPEG-PDPA hardly affected the expression of these proteins (*p* > 0.05). These results indicated that in the *in vivo* experiments, 1) mPEG-PDPA hardly affects the development of osteosarcoma, and 2) FOXD2-AS1 promotes the development of osteosarcoma mediated by the COI-1/OCN/Runx-2 signaling pathway.

**Figure 4:**
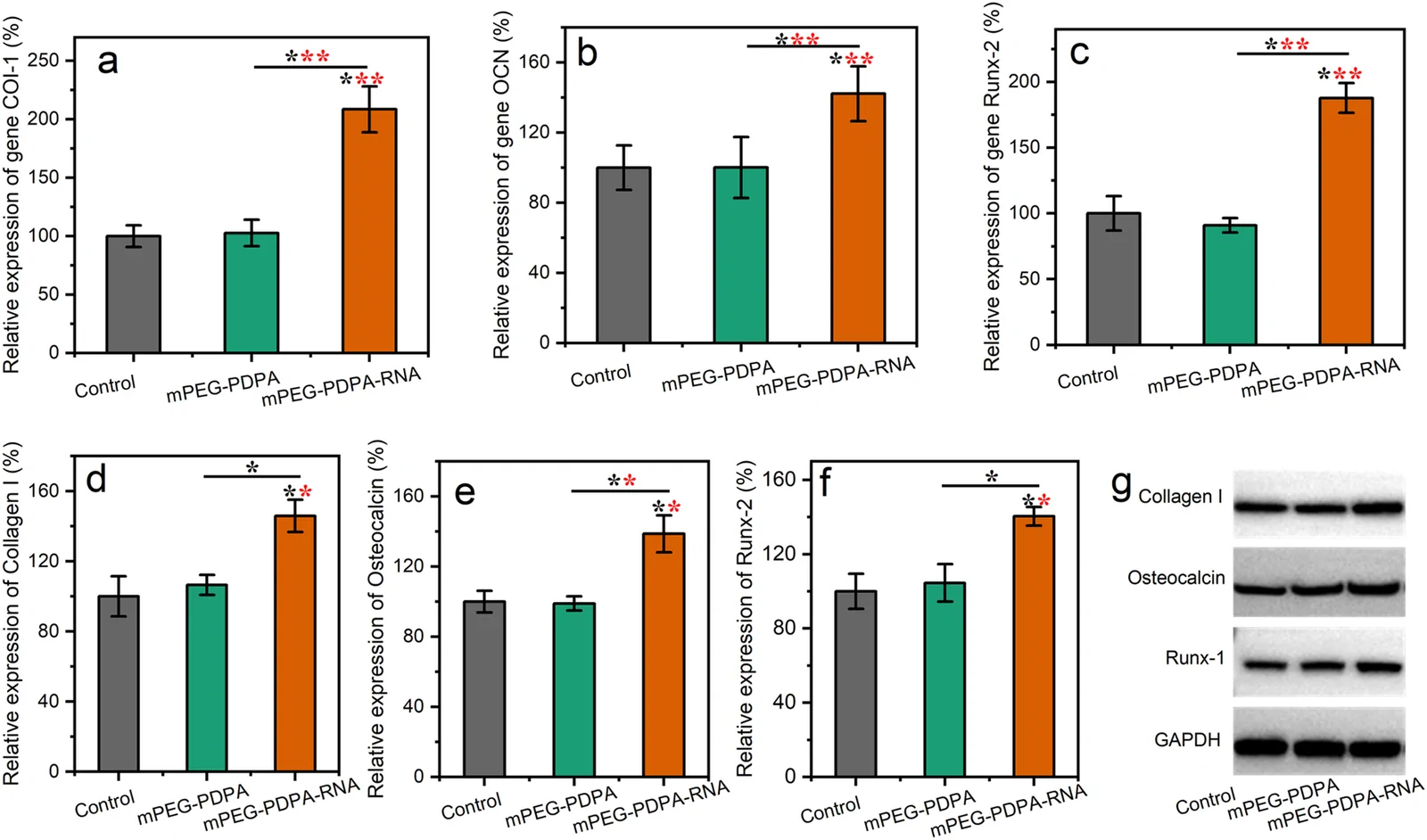
Exploration of the molecular mechanisms by which FOXD2-AS1 regulates osteosarcoma development. Relative expression of **(a)** COI-1, **(b)** OCN and **(c)** Runx-2 genes (*n* = 6). Relative expression of **(d)** Collagen I, **(e)** Osteocalcin and **(f)** Runx-2 proteins, and **(g)** corresponding Western blot (WB) bands, where full-length WB bands are given in Supplementary Figure S15 (*n* = 3). *, ** and *** represent the results of statistical difference analysis with *p*-values less than 0.05, 0.01 and 0.001, respectively. The red and black stars in the figures represent the same statistical significance levels, with the color difference serving only as a visual aid to highlight the increasing levels of significance.

Our mechanistic investigation, moving from RNA-seq screening to targeted RT-qPCR and Western blot validation, identified and confirmed the activation of the COI-1/OCN/Runx-2 osteogenic signaling axis as a key event following FOXD2-AS1 silencing. The concordance between the transcriptomic data and the orthogonal validation by RT-qPCR strengthens the reliability of this finding.

### 3.5 Functional differences under FOXD2-AS1 mediation

Differences in gene function mediated by FOXD2-AS1 were revealed through the transcriptome. As shown in Figure 5a, Gene Ontology (GO) analysis revealed that leukocyte migration, cell-substrate adhesion and cell chemotaxis were the major differential biological processes (BP), collagen-containing extracellular matrix, focal adhesion and cell-substrate junction were the major differential cellular components (CC), while integrin binding, virus receptor activity and exogenous protein binding are the major differential molecular functions (MF). Furthermore, Kyoto encyclopedia of genes and genomes (KEGG) enrichment analysis revealed that functional genes under the influence of FOXD2-AS1 were enriched at focal adhesion, rap1 signaling pathway, apoptosis and cell cycle (Figure 5b). These results demonstrated that FOXD2-AS1 regulates osteosarcoma metastasis as well as progression through its effects on biological processes such as cell migration, apoptosis and cell cycle. Moreover, Empowering the development of genomics expertise (EDGE) analyze results revealed potential genes significantly associated with osteosarcoma development mediated by FOXD2-AS1: CPS1-IT1, ANKRD10-IT1, TBC1D3P1-DHX40P1, and RGS5 (Supplementary Figure S16). These results suggested that FOXD2-AS1 regulates CPS1-IT1, ANKRD10-IT1, TBC1D3P1-DHX40P1 and RGS5 expression thereby further affecting osteosarcoma metastasis and progression.

**Figure 5:**
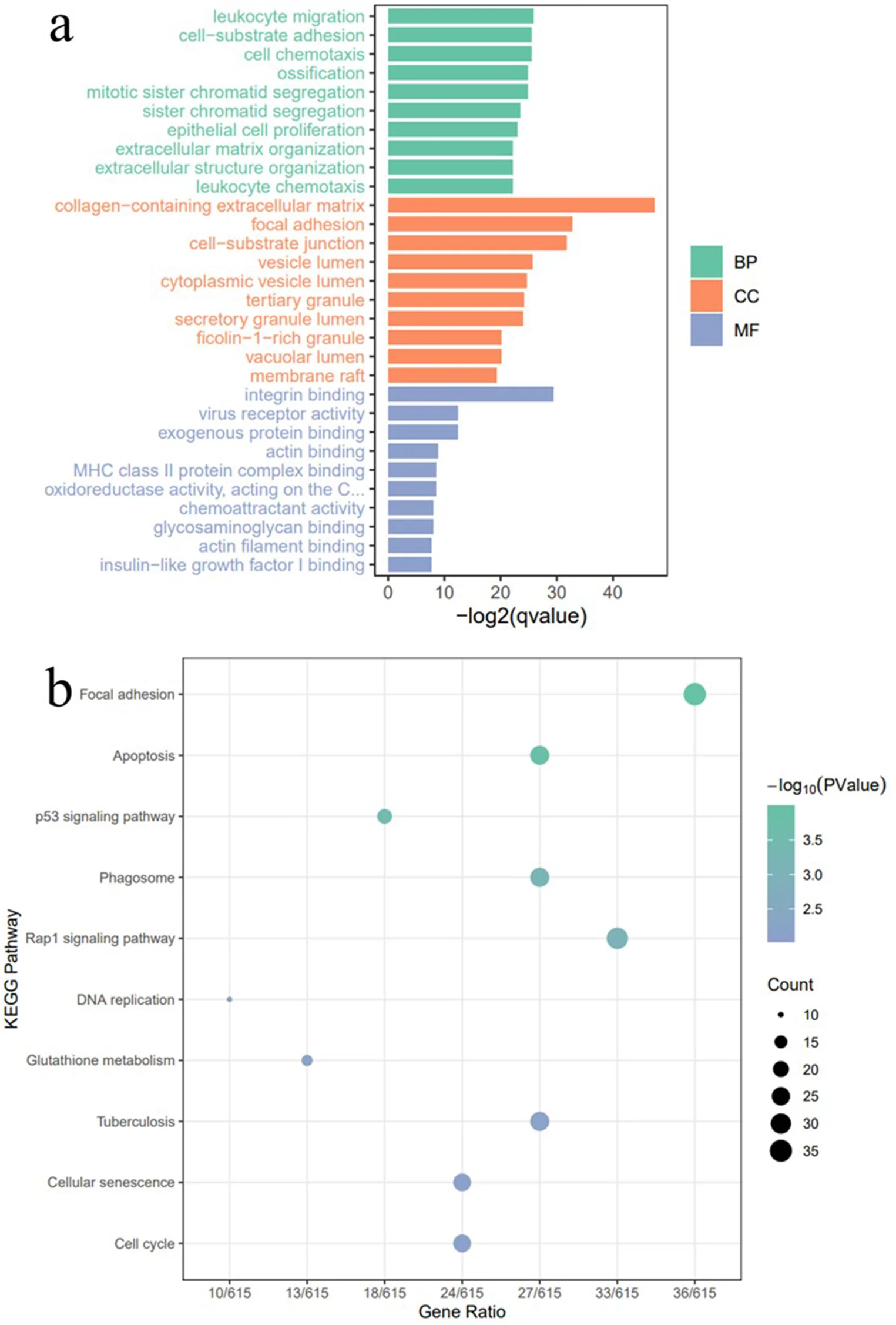
Transcriptomics reveals functional differences under FOXD2-AS1-mediated (*n* = 3). **(a)** Results of gene ontology analysis. **(b)** Results of Kyoto encyclopedia of genes and genomes analysis.

## 4 Discussion

Nowadays, increasing studies have revealed the important regulatory role of LncRNAs in tumor development and malignancy (33, 34). FOXD2-AS1 is a LncRNA of 2,527 bp in a cluster of genes located in the telomere region of the human chromosome at the 1p33 position (16). Several existing studies have shown that LncRNA FOXD2-AS1 is aberrantly expressed in cells of various tumor tissues (24, 35–37). Highly bio-affinity hydrogels can transfer functional RNAs in cell-based therapies, which offers great promise for tumor therapy (29). Our study designed engineered hydrogels as delivery vehicles for targeting LncRNA FOXD2-AS1. We packaged the highly expressed LncRNA FOXD2-AS1 into mPEG-PDPA hydrogels and applied mPEG-PDPA-RNA to osteosarcoma cells. After preparation of mPEG-PDPA-RNA, we investigated its targeting ability and observed the effective tumor targeting ability of mPEG-PDPA-RNA *in vitro* and *in vivo*.

In this study, we synthesized mPEG-PDPA hydrogel by free radical polymerization reaction and used it as a delivery vehicle for LncRNA FOXD2-AS1. FTIR spectroscopy analysis revealed that -OH was the main active site for mPEG-PDPA binding to FOXD2-AS1, while DLS tests confirmed the stability of mPEG-PDPA-RNA. This is due to the fact that the hydrogel surface is rich in hydrophilic groups, while the bases of RNA are hydrophilic, and the two are highly susceptible to binding by hydrogen bonding and van der Waals interactions (38). This was also confirmed in the FTIR spectra of mPEG-PDPA-RNA due to the appearance of IR peaks attributed to the bases.

Furthermore, this study reveals FOXD2-AS1 as a core multifunctional regulator in osteosarcoma progression. Its knockdown not only directly suppresses malignant phenotypes by inhibiting proliferation (downregulation of CCK-8 and Ki67) and migration (wound healing assay), but also orchestrates broad programmatic reprogramming at the molecular level. It activates the COI-1/OCN/Runx2 osteogenic signaling axis while simultaneously driving stemness (upregulation of Nanog, OCT4, SOX2, CD133) and epithelial-mesenchymal transition (upregulation of N-cadherin, Vimentin, Snail; downregulation of E-cadherin), thereby maintaining tumor cell aggressiveness and plasticity. Furthermore, its regulation of VEGF and MMPs further modulates angiogenesis and extracellular matrix degradation. Thus, FOXD2-AS1 integrates multiple pathways—proliferation, migration, stemness, and EMT—to collectively drive osteosarcoma progression. The mPEG-PDPA hydrogel-based delivery system, by efficiently silencing this hub molecule, simultaneously disrupts the above oncogenic network, offering a novel strategy for multi-target combination therapy.

The mechanism of action of mPEG-PDPA-RNA on osteosarcoma cells and tissues was explored by *in vivo* and *in vitro* experiments. Cellular experiments showed that the pristine mPEG-PDPA hydrogel was non-toxic and an excellent carrier material with high bio-affinity. *In vivo* experiments showed that mPEG-PDPA-RNA delivery of FOXD2-AS1 resulted in elevated alkaline phosphatase (ALP) levels and up-regulated ALP gene expression in osteosarcoma model mice. Moreover, FOXD2-AS1 induced the development of inflammatory responses in osteosarcoma tissues. In addition, immunohistochemistry showed that FOXD2-AS1 inhibited the expression of CD31 and Ki67 in osteosarcoma tissues. RT-qPCR, Western blot and histological examination revealed that activation of the COI-1/OCN/Runx-2 signaling pathway promoted the deterioration of osteosarcoma, which involves a number of biological processes such as cell migration, apoptosis and cell cycle. In addition, genes such as CPS1-IT1, ANKRD10-IT1, TBC1D3P1-DHX40P1, and RGS5 were shown to play important interactions in FOXD2-AS1-induced osteosarcoma deterioration. Thus, LncRNA FOXD2-AS1 may be a promising therapeutic target for OS inhibition.

To molecularly validate the pro-osteogenic phenotype suggested by the elevated serum ALP and to confirm key findings from our transcriptomic analysis, we performed RT-qPCR on tumor tissues. Consistent with the functional assay, ALP gene expression was significantly up-regulated in the mPEG-PDPA-RNA group compared to controls (Figure 3b). Furthermore, targeted analysis of the osteogenic differentiation pathway, which was highlighted in our RNA-seq data, revealed a significant transcriptional upregulation of its core components: including COI-1, OCN, and the master regulator Runx-2 (Figures 4a–c). These RT-qPCR results provided direct confirmation of the specific transcriptomic changes induced by FOXD2-AS1 knockdown and guided our subsequent focus on the COI-1/OCN/Runx-2 axis for protein-level validation.

Serum alkaline phosphatase (ALP) is significantly elevated in patients with osteosarcoma compared to healthy individuals and is associated with osteosarcoma (39). Our results showed that ALP levels in osteosarcoma tissues of model rats were significantly upregulated after mPEG-PDPA-RNA treatment. It indicated that FOXD2-AS1 induces ALP production in osteosarcoma tissues. Similar to previous reports (37, 40), our results suggest that FOXD2-AS1 induces ALP production and inflammatory response in osteosarcoma tissues with a definite role in worsening osteosarcoma. Zhang et al. reported that knockdown of FOXD2-AS1 promotes apoptosis and impairs the invasive and migratory abilities of osteosarcoma cells (37). FOXD2-AS1 significantly inhibited the expression of the tumor angiogenesis biomarker CD31 and the cell proliferation biomarker Ki67 in osteosarcoma tissues. This indicated that the promotion of osteosarcoma progression by FOXD2-AS1 was not achieved through cell proliferation and tumor angiogenesis pathways (41). Activation of the osteogenic protein COI-1/OCN/Runx-2 signaling pathway suggests that FOXD2-AS1 induces transitional activation of osteoblasts. The osteoblastic phenotype is the main subtype classification of the major osteosarcomas, suggesting that the acceleration of osteosarcoma progression by FOXD2-AS1 may originate from the promotion of malignant transformation of bone mesenchymal cells (1, 42).

The transcriptomics results confirmed that cell-substrate adhesion, cell chemotaxis and exogenous protein binding were the main differential processes when FOXD2-AS1 induced the deterioration of osteosarcoma, while cell migration, apoptosis and cell cycle were the mainly affected biological processes. This further suggested that regulating the transitional activation of osteoblasts and activating the COI-1/OCN/Runx-2 signaling pathway, which induces malignant transformation of bone mesenchymal stromal cells, are potential mechanisms by which FOXD2-AS1 deteriorates osteosarcoma. The expression of CPS1-IT1, ANKRD10-IT1, TBC1D3P1-DHX40P1 and RGS5 genes would be involved during this period.

## 5 Conclusion

In conclusion, our results revealed that mPEG-PDPA hydrogels can serve as excellent LncRNA FOXD2-AS1 delivery vehicles with virtually no toxic side effects *in vivo* and *in vitro*. Moreover, FOXD2-AS1 worsened osteosarcoma in model mice, leading to upregulation of ALP and inflammatory responses. Mechanistic investigations revealed that activation of the COI-1/OCN/Runx-2 signaling pathway and subsequent regulation of biological processes such as cell migration, apoptosis and cell cycle are important mechanisms by which FOXD2-AS1 promotes osteosarcoma. Our results provided new insights into the pathogenesis of osteosarcoma and provided valuable experience for subsequent pathological studies and the development of corresponding therapeutic strategies.

## Data Availability

The Omics-related data presented in the study are deposited in the Science Data Bank repository, accession Link: https://www.scidb.cn/s/yAVzii. Other original contributions presented in the study are included in the Supplementary Material. Further inquiries can be directed to the corresponding author.

## References

[1] Gill J Gorlick R. Advancing therapy for osteosarcoma. Nat Rev Clin Oncol. (2021) 18(10):609–24. 10.1038/s41571-021-00519-8 34131316

[2] Beird HC Bielack SS Flanagan AM Gill J Heymann D Janeway KA. Osteosarcoma. Nat Rev Dis Primers. (2022) 8(1):77. 10.1038/s41572-022-00409-y 36481668

[3] Meltzer PS Helman LJ. New horizons in the treatment of osteosarcoma. N Engl J Med. (2021) 385(22):2066–76. 10.1056/NEJMra2103423 34818481

[4] Tian H Cao J Li B Nice EC Mao H Zhang Y. Managing the immune microenvironment of osteosarcoma: the outlook for osteosarcoma treatment. Bone Res. (2023) 11(1):11. 10.1038/s41413-023-00246-z 36849442 PMC9971189

[5] Jiang Y Wang J Sun M Zuo D Wang H Shen J. Multi-omics analysis identifies osteosarcoma subtypes with distinct prognosis indicating stratified treatment. Nat Commun. (2022) 13(1):7207. 10.1038/s41467-022-34689-5 36418292 PMC9684515

[6] Kafita D Daka V Nkhoma P Zulu M Zulu E Tembo R. High ELF4 expression in human cancers is associated with worse disease outcomes and increased resistance to anticancer drugs. PLoS One. (2021) 16(4):e0248984. 10.1371/journal.pone.0248984 33836003 PMC8034723

[7] Sinkala M. Mutational landscape of cancer-driver genes across human cancers. Sci Rep. (2023) 13(1):12742. 10.1038/s41598-023-39608-2 37550388 PMC10406856

[8] Pang J Huang L Lian Y Yuan Z Wang F Zhang L-M. Co-delivery of siAEG-1 and doxorubicin to treat osteosarcoma via nanomicelles for azide−alkyne “click” conjugation of poly(l-lysine) dendrons onto Zein. Int J Biol Macromol. (2024) 264:130729. 10.1016/j.ijbiomac.2024.130729 38460643

[9] Amit M Takahashi H Dragomir MP Lindemann A Gleber-Netto FO Pickering CR. Loss of p53 drives neuron reprogramming in head and neck cancer. Nature. (2020) 578(7795):449–54. 10.1038/s41586-020-1996-3 32051587 PMC9723538

[10] Abdul-Rahman T Lizano-Jubert I Bliss ZSB Garg N Meale E Roy P. RNA In cardiovascular disease: a new frontier of personalized medicine. Prog Cardiovasc Dis. (2024) 85:93–102. 10.1016/j.pcad.2024.01.016 38253161

[11] Liu W-W Zheng S-Q Li T Fei Y-F Wang C Zhang S. RNA Modifications in cellular metabolism: implications for metabolism-targeted therapy and immunotherapy. Signal Transduction Targeted Ther. (2024) 9(1):70. 10.1038/s41392-024-01777-5 PMC10966055 38531882

[12] Arab I Park J Shin J-J Shin H-S Suk K Lee W-H. Macrophage lncRNAs in cancer development: long-awaited therapeutic targets. Biochem Pharmacol. (2023) 218:115890. 10.1016/j.bcp.2023.115890 37884197

[13] Wang P Chen Y Zheng Y Fu Y Ding Z. Identification of epithelial-mesenchymal transition- (EMT-) related LncRNA for prognostic prediction and risk stratification in esophageal squamous cell carcinoma. Dis Markers. (2021) 2021:5340240. 10.1155/2021/5340240 34712369 PMC8548124

[14] Wang P Zhu J Long Q Wang Y Xu H Tao H. LncRNA SATB2-AS1 promotes tumor growth and metastasis and affects the tumor immune microenvironment in osteosarcoma by regulating SATB2. J Bone Oncol. (2023) 41:100491. 10.1016/j.jbo.2023.100491 37601080 PMC10436287

[15] Li R Chen S Zhan J Li X Liu W Sheng X. Long noncoding RNA FOXD2-AS1 enhances chemotherapeutic resistance of laryngeal squamous cell carcinoma via STAT3 activation. Cell Death Dis. (2020) 11(1):41. 10.1038/s41419-020-2232-7 31959918 PMC6971019

[16] Ghafouri-Fard S Harsij A Hussen BM Pourmoshtagh H Taheri M. A review on the role of FOXD2-AS1 in human disorders. Pathol Res Pract. (2024) 254:155101. 10.1016/j.prp.2024.155101 38211387

[17] Ren Z Hu Y Li G Kang Y Liu Y Zhao H. HIF-1*α* induced long noncoding RNA FOXD2-AS1 promotes the osteosarcoma through repressing p21. Biomed Pharmacother. (2019) 117:109104. 10.1016/j.biopha.2019.109104 31228799

[18] An X Yang J Cui X Zhao J Jiang C Tang M. Advances in local drug delivery technologies for improved rheumatoid arthritis therapy. Adv Drug Delivery Rev. (2024) 209:115325. 10.1016/j.addr.2024.115325 38670229

[19] Ashique S Faiyazuddin M Afzal O Gowri S Hussain A Mishra N. Advanced nanoparticles, the hallmark of targeted drug delivery for osteosarcoma-an updated review. J Drug Delivery Sci Technol. (2023) 87:104753. 10.1016/j.jddst.2023.104753

[20] Liu H Guo Z Ma W Li S Wang D Zheng Z. UV Irradiation-Induced cyclic-to-linear topological changes of a light/pH dual-sensitive cyclic copolymer for enhanced drug delivery. ACS Macro Lett. (2023) 12(8):1025–30. 10.1021/acsmacrolett.3c00265 37432938

[21] Papadimitriou L Theodorou A Papageorgiou M Voutyritsa E Papagiannaki A Velonia K. Ranella, pH responsive biohybrid BSA-poly(DPA) nanoparticles for interlysosomal drug delivery. J Drug Delivery Sci Technol. (2022) 75:103591. 10.1016/j.jddst.2022.103591

[22] Dong Y Zhou J Wang C Wang Y Deng L Zhang J. Comb-like amphiphilic polycarbonates with different lengths of cationic branches for enhanced siRNA delivery. Macromol Biosci. (2020) 20(7):2000143. 10.1002/mabi.202000143 32401412

[23] Li P Shi G Zhang X Song H Zhang C Wang W. Guanidinylated cationic nanoparticles as robust protein antigen delivery systems and adjuvants for promoting antigen-specific immune responses *in vivo*. J Mater Chem B. (2016) 4(33):5608–20. 10.1039/C6TB01556E 32263357

[24] Ren Z Hu Y Sun J Kang Y Li G Zhao H. N6-methyladenosine methyltransferase WTAP-stabilized FOXD2-AS1 promotes the osteosarcoma progression through m6A/FOXM1 axis. Bioengineered. (2022) 13(4):7963–73. 10.1080/21655979.2021.2008218 35356854 PMC9161975

[25] Contag CH Bachmann MH. Advances in *in vivo* bioluminescence imaging of gene expression. Annu Rev Biomed Eng. (2002) 4:235–60. 10.1146/annurev.bioeng.4.111901.093336 12117758

[26] Wang Y Lin H Huang W Liu Z Chen Z Zhao X. Curcumin attenuates periodontal injury via inhibiting ferroptosis of ligature-induced periodontitis in mice. Int J Mol Sci. (2023) 24(12):9835. 10.3390/ijms24129835 37372983 PMC10298010

[27] Luan G Wang M Yuan J Bu X Song J Wang C. Regulatory network identified by pulmonary transcriptome and proteome profiling reveals extensive change of tumor-related genes in microRNA-21 knockout mice. J Cancer Res Clin Oncol. (2022) 148(8):1919–29. 10.1007/s00432-022-03967-6 35511299 PMC11801160

[28] Kitane DL Loukman S Marchoudi N Fernandez-Galiana A El Ansari FZ Jouali F. A simple and fast spectroscopy-based technique for COVID-19 diagnosis. Sci Rep. (2021) 11(1):16740. 10.1038/s41598-021-95568-5 34408169 PMC8373901

[29] Zhong R Talebian S Mendes BB Wallace G Langer R Conde J. Hydrogels for RNA delivery. Nat Mater. (2023) 22(7):818–31. 10.1038/s41563-023-01472-w 36941391 PMC10330049

[30] Lei Y Huang S Sharif-Kashani P Chen Y Kavehpour P Segura T. Incorporation of active DNA/cationic polymer polyplexes into hydrogel scaffolds. Biomaterials. (2010) 31(34):9106–16. 10.1016/j.biomaterials.2010.08.016 20822811 PMC3017740

[31] Bhatti Z Kiran K Shoaib M Nisa SU Arif A. Alkaline phosphatase as serum tumor marker in osteosarcoma. Pak J Med Health Sci. (2022) 16(3):30. 10.53350/pjmhs2216330

[32] Barger A Baker K Driskell E Sander W Roady P Berry M. The use of alkaline phosphatase and runx2 to distinguish osteosarcoma from other common malignant primary bone tumors in dogs. Vet Pathol. (2022) 59(3):427–32. 10.1177/03009858221083035 35253545

[33] Yuan Y Yu P Shen H Xing G Li W. LncRNA FOXD2-AS1 increased proliferation and invasion of lung adenocarcinoma via cell-cycle regulation. Pharmgenomics Pers Med. (2023) 16:99–109. 10.2147/pgpm.s396866 36761100 PMC9904230

[34] Park EG Pyo SJ Cui Y Yoon SH Nam JW. Tumor immune microenvironment lncRNAs. Brief Bioinform. (2022) 23(1):bbab504. 10.1093/bib/bbab504 34891154 PMC8769899

[35] Wang Y Cheng Y Yang Q Kuang L Liu G. Overexpression of FOXD2-AS1 enhances proliferation and impairs differentiation of glioma stem cells by activating the NOTCH pathway via TAF-1. J Cell Mol Med. (2022) 26(9):2620–32. 10.1111/jcmm.17268 35419917 PMC9077300

[36] Duan F Li H Liu W Zhao J Yang Z Zhang J. Long non-coding RNA FOXD2-AS1 serves as a potential prognostic biomarker for patients with cancer: a meta-analysis and database testing. Am J Med Sci. (2021) 362(2):173–81. 10.1016/j.amjms.2021.01.020 34303519

[37] Zhang QQ Xu SL Ding C Ma CC Yuan TS Hua CC. LncRNA FOXD2-AS1 knockdown inhibits the resistance of human osteosarcoma cells to cisplatin by inhibiting miR-143 expression. Eur Rev Med Pharmacol Sci. (2021) 25(2):678–86. 10.26355/eurrev_202101_24629 33577022

[38] Li J Zhang P Zhou M Liu C Huang Y Li L. Trauma-responsive scaffold synchronizing oncolysis immunization and inflammation alleviation for post-operative suppression of cancer metastasis. ACS Nano. (2022) 16(4):6064–79. 10.1021/acsnano.1c11562 35344338

[39] Agustina H Asyifa I Aziz A Hernowo BS. The role of osteocalcin and alkaline phosphatase immunohistochemistry in osteosarcoma diagnosis. Patholog Res Int. (2018) 2018:6346409. 10.1155/2018/6346409 29854380 PMC5960576

[40] Han J Shen X. Long noncoding RNAs in osteosarcoma via various signaling pathways. J Clin Lab Anal. (2020) 34(6):e23317. 10.1002/jcla.23317 32249459 PMC7307344

[41] Razavi SM Yahyaabadi R. Comparative study of correlation between angiogenesis markers (CD31) and Ki67 marker with behavior of aggressive and nonaggressive central giant cell granuloma with immunohistochemistry technique. Asian Pac J Cancer Prev. (2018) 19(8):2279–83. 10.22034/apjcp.2018.19.8.2279 30139237 PMC6171410

[42] Fang Y Yu Y Jiang X Liu P Chen Y Feng W. Self-adaptive MoO3−x subnanometric wires incorporated scaffolds for osteosarcoma therapy and bone regeneration. Adv Funct Mater. (2023) 33(49):2304163. 10.1002/adfm.202304163

