# Supplementary material for: Pathological mechanisms of long non-coding RNA FOXD2-AS1 promoting osteosarcoma mediated by the COI-1/OCN/Runx-2 signaling pathway
Source: Front Med (Lausanne). 2026 Sep 30;13:1664094. doi: 10.3389/fmed.2026.1664094 (PMC13661506; doi:10.3389/fmed.2026.1664094)
Supplement: Supplementary file 1 [file Table1.docx]

**Pathological mechanisms of long non-coding RNA FOXD2-AS1 promoting osteosarcoma mediated by the COI-1/OCN/Runx-2 signaling pathway**

[Zhipeng Ren](https://pubmed.ncbi.nlm.nih.gov/?term=Ren+Z&cauthor_id=38665015)^1^, [Liandong Deng](https://pubmed.ncbi.nlm.nih.gov/?term=Deng+LD&cauthor_id=17577635)^2^, [Jie Sun](https://pubmed.ncbi.nlm.nih.gov/?term=Sun+J&cauthor_id=38665015)^1^, Yuxiang Kang^1^, [Hejun Zhao](https://pubmed.ncbi.nlm.nih.gov/?term=Zhao+H&cauthor_id=38665015)^3*^

*^1^ Department of Orthopaedics, Tianjin Hospital, 300211 Tianjin, China.*

*^2^ Department of Polymer Science and Technology, School of Chemical Engineering and Technology, Tianjin University, Tianjin 300072, China.*

*^3^ Department of Endocrinology and Metabolism, Tianjin First Central Hospital, 300192 Tianjin, China.*

* Corresponding authors: zhaohejun1985@126.com

[1. Supplementary Text (1)	2](#_Toc165749525)

[2. Supplementary Table (3)	3](#_Toc165749526)

[3. Supplementary Figure (16)	6](#_Toc165749527)

## 1. Supplementary Text

**Text S1. Synthesis of mPEG-PDPA.**

Firstly, 0.2 g of mPEG, 0.605 mL of DPA, 0.015 g of AIBN and 15 mL of DMF were added into the Schlenk tube, and the system was evacuated for 15 min and filled with nitrogen until the system was at atmospheric pressure. The system was heated and stirred at 73 ℃ for 7 h. Afterwards, the reaction solution was transferred to a 1000 kDa dialysis bag and dialyzed in deionized water for 72 h, during which the deionized water was replaced every 6 h to remove unreacted precursors. After dialysis, the reaction solution was freeze-dried to obtain the amphiphilic polymer mPEG-PDPA.

## 2. Supplementary Table

**Table S1.** Sources and dilution parameters of primary and secondary antibodies used in immunohistochemistry assay.

| Antibody | Proteins | Source | Manufacturer | No. | Dilution ratio |
| --- | --- | --- | --- | --- | --- |
| Primary antibody | CD31 | Rabbit | zsbio | ZA-0568 | 1:200 |
|  | Ki67 | Rabbit | abcam | ab16667 | 1:200 |
| Secondary antibody | Goat Anti-Rabbit IgG Secondary Antibody (HRP) | Goat | Sino biological | SSA004 | 1:1500 |

**Table S2.** Summary of primer sequences used in RT-qPCR analysis.

| Gene |  | Primer sequences (5’-3’) | Size (bp) |
| --- | --- | --- | --- |
| ALP | F | CCTTACCAACTCTTTTGTGCCAG | 165 |
|  | R | GTTACTGTGGAGACGCCCATACC |  |
| COl-1 | F | AAACTTTGCTTCCCAGATGTCC | 178 |
|  | R | CATTGGACCTGAACCGCC |  |
| Runx-2 | F | GAATGATGAGAACTACTCCGCCG | 112 |
|  | R | ACTCTTGCCTCGTCCGCTCC |  |
| OCN | F | ACCATCTTTCTGCTCACTCTGCTG | 127 |
|  | R | TCACTACCTTATTGCCCTCCTGC |  |
| GAPDH | F | CGGTGCTGAGTATGTCGTGGAGTC | 100 |
|  | R | GGCGGAGATGATGACCCTTTTG |  |

**Table S3.** Sources and dilution parameters of primary and secondary antibodies used in the western blot (WB) assay.

| Antibody | Proteins | Source | Manufacturer | No. | Dilution ratio |
| --- | --- | --- | --- | --- | --- |
| Primary antibody | Collagen I | Rabbit | CST | #91144 | 1:800 |
|  | Osteocalcin | Rabbit | CST | #59757 | 1:1000 |
|  | Runx2 | Rabbit | CST | #12556 | 1:1000 |
|  | GAPDH | Mouse | Utibody | UM4002 | 1:2000 |
| Secondary antibody | Goat anti-rabbit lgG-HRP | Goat | Affinity | bs-0295G-HRP | 1:4000 |
|  | Goat anti-mouse lgG-HRP | Goat | Affinity | bs-0296G-HRP | 1:4000 |

## 3. Supplementary Figure

**Figure S1.** Screening for the most stable mPEG-PDPA-RNA material: (a) changes in zeta potential, (b) changes in size.

**Figure S2.** Cumulative release profile of mPEG-DPA-1/60.

**Figure S3.** Representative results of flow apoptosis assay (n = 3): the effect of 24 h survival of MG63 osteosarcoma cells at different mPEG-PDPA doses.

**Figure S4.** Representative results of flow cell cycle assay (n = 3): the effect of mPEG-PDPA doses on MG63 osteosarcoma cells cycle.

**Figure S5.** BCA analysis for (a) ALP content testing in osteosarcoma and (b) WB testing, n = 6.

**Figure S6.** Representative immunohistochemistry plots of (a-c) CD31 and (d-f) Ki67, n = 6.

**Figure S7.** Changes in tumor volume in nude mice (n = 6).

**Figure S8.** *In vivo* imaging of tumor growth in nude mice.

**Figure S9.** Representative photographs of tumor-bearing mice: (a) Control group, (b) mPEG-PDPA and (c) mPEG-PDPA-RNA.

**Figure S10.** Cell viability was assessed using the CCK-8 assay in human osteosarcoma MG-63 cells cultured in their specific growth medium.

**Figure S11.** Cell migration was assessed by a wound healing (scratch) assay in MG-63 human osteosarcoma cells.

**Figure S12.** Cell migration was evaluated using a Transwell migration assay with MG-63 human osteosarcoma cells (200 ×).

**Figure S13.** Western blot analysis.

**Figure S14.** Optical images of western blot for stemness, EMT, metastasis and angiogenesis proteins. GAPDH was used as loading control.

**Figure S15.** Full-length WB bands (from left to right: Control, mPEG-PDPA, and mPEG-PDPA-RNA groups), with the number of parallel groups being 3.

**Figure S16.** Empowering the development of genomics expertise analyze result.
